# CHANGES IN MULTIMORBIDITY AMONG HOSPITALIZED ADULTS IN THE US

**DOI:** 10.1093/geroni/igad104.3217

**Published:** 2023-12-21

**Authors:** Lauren Picken, Yue Zhang, Christine Loyd

**Affiliations:** University of Alabama at Birmingham, Birmingham, Alabama, United States; University of Alabama at Birmingham, Birmingham, Alabama, United States; University of Alabama at Birmingham, Birmingham, Alabama, United States

## Abstract

Having multiple chronic health conditions, or multimorbidity, is common among adult inpatients and is associated with risk of death, disability, and reduced quality of life. This investigation examines prevalence trends of multimorbidity over time among inpatients based on age, sex, and race and assesses hospitalization rates for individual diseases over time. Retrospective cross-sectional analysis of 2012-2018 US National Inpatient Sample datasets was completed. Participants were hospitalized patients ≥55y from community hospitals. ICD-9 and 10 codes for admitting diagnoses were used to calculate disease burden using the Charlson Comorbidity Index (CCI) and Elixhauser Comorbidity Index (ECI). Unweighted mean index scores and admission rates for diseases were compared. Mean age for the sample was 72y, and inpatients were mostly female (53%) and White (77%). The mean comorbidity scores increased across the sample: mean CCI increased from 1.69 to 1.97 and mean ECI increased from 3.66 to 4.14. Comorbidity scores were higher over time with increasing age until about age 80-84 (for CCI) and age 85-89 (for ECI) and increased similarly among males and females. Black and Native American inpatients had the largest increase in mean CCI and ECI scores. Comorbidities with increased hospitalization rates included congestive heart failure (+5%), dementia (+6%), complicated diabetes (+14%), complicated hypertension (+17%), renal disease/failure (+5%), and obesity (+5%). Growing disease burden among inpatients supports the continued need for programs aiming to prevent and treat chronic diseases and multimorbidity, especially among underrepresented populations including Black and Native American communities.

